# The Safety and Efficacy of Phage Therapy for Superficial Bacterial Infections: A Systematic Review

**DOI:** 10.3390/antibiotics9110754

**Published:** 2020-10-29

**Authors:** Angharad Steele, Helen J. Stacey, Steven de Soir, Joshua D. Jones

**Affiliations:** 1 Infection Medicine, Edinburgh Medical School: Biomedical Sciences, University of Edinburgh, Chancellor’s Building, 49 Little France Crescent, Edinburgh EH16 4SB, UK; ani.steele@gmail.com; 2 Edinburgh Medical School, University of Edinburgh, Chancellor’s Building, 49 Little France Crescent, Edinburgh EH16 4SB, UK; s1204283@sms.ed.ac.uk; 3 Laboratory for Molecular and Cellular Technology, Queen Astrid Military Hospital, Rue Bruyn, 1120 Brussels, Belgium; steven.desoir@uclouvain.be; 4 Cellular & Molecular Pharmacology, Louvain Drug Research Institute, Université Catholique de Louvain (UCLouvain), avenue E. Mounier 73, 1200 Brussels, Belgium

**Keywords:** burn wound infection, chronic wound infection, diabetic foot infection, phage therapy, superficial infection, systematic review

## Abstract

Superficial bacterial infections, such as dermatological, burn wound and chronic wound/ulcer infections, place great human and financial burdens on health systems globally and are often complicated by antibiotic resistance. Bacteriophage (phage) therapy is a promising alternative antimicrobial strategy with a 100-year history of successful application. Here, we report a systematic review of the safety and efficacy of phage therapy for the treatment of superficial bacterial infections. Three electronic databases were systematically searched for articles that reported primary data about human phage therapy for dermatological, burn wound or chronic wound/ulcer infections secondary to commonly causative bacteria. Two authors independently assessed study eligibility and performed data extraction. Of the 27 eligible reports, eight contained data on burn wound infection (*n* = 156), 12 on chronic wound/ulcer infection (*n* = 327) and 10 on dermatological infections (*n* = 1096). Cautionary pooled efficacy estimates from the studies that clearly reported efficacy data showed clinical resolution or improvement in 77.5% (*n* = 111) of burn wound infections, 86.1% (*n* = 310) of chronic wound/ulcer infections and 94.14% (*n* = 734) of dermatological infections. Over half of the reports that commented on safety (*n* = 8/15), all published in or after 2002, did not express safety concerns. Seven early reports (1929–1987), described adverse effects consistent with the administration of raw phage lysate and co-administered bacterial debris or broth. This review strongly suggests that the use of purified phage to treat superficial bacterial infections can be highly effective and, by various routes of administration, is safe and without adverse effects.

## 1. Introduction

The antibiotic resistance crisis threatens to return medicine to the pre-antibiotic era and is predicted to cost up to $100 trillion and lead to an annual 10 million premature deaths by 2050 [1,2]. The effects of this crisis are compounded by a decline in the number of new antibiotics brought to market, driven by a range of factors that discourage investment [2]. This synergy has stimulated an increase in the research and development of novel antimicrobials.

Bacteriophage (phage) are viruses of bacteria, independently discovered in 1915 and 1917 [3]. The species, and often even strain, specific manner of lytic phages can be exploited for antibacterial therapy, known as phage therapy [4]. Phage therapy was widely used in the 1920s but various factors, including the introduction of antibiotics in the 1940s, led to its demise in the West [3,5]. Despite this, phage therapy has remained commonplace in Eastern Europe and the former Soviet Union, with institutions such as Eliava (Tbilisi, Georgia) and Hirszfeld (Wroclaw, Poland) continuing to carry out phage production, research and therapy [5].

Phage therapy is experiencing a renaissance in the geopolitical West, catalysed by the high-profile successful treatment of a patient in 2017 with a systemic multi-drug resistant *Acinetobacter* infection in the United States (US) using intravenous and intraperitoneal phage [6]. Subsequently, other successful cases of intravenous phage therapy have also been reported [7,8,9]. Alongside life-threatening systemic disease there is great interest in the use of phage therapy for superficial bacterial infections, in particular dermatological, burn and chronic wound/ulcer infections [10,11,12]. In such cases phage therapy can be applied topically, administered orally or injected subcutaneously [10,12,13]. Based on bacterial susceptibility screening, phage therapy can be administered using standardised pre-prepared formulations or bespoke formulations containing one or more phage(s) [14].

The American Academy of Dermatology estimates that in 2013, 17.4 million Americans received treatment for burns, ulcers or wounds; while 40.8 million sought treatment for any form of skin infection. The cost of treating skin infections was estimated to be $8.1 billion, while the treatment of burns and wounds cost approximately $6.4 billion and ulcers approximately $5.4 billion [15,16]. These conditions are often complicated by the presence of bacteria that exhibit antibiotic resistance [17,18]. A wide variety of bacterial pathogens are found in these conditions, which span community and hospital settings. Among skin and soft tissue infections, the SENTRY antimicrobial surveillance programme has identified *Staphylococcus aureus*, *Pseudomonas aeruginosa*, *Enterococcus* spp. and *Escherichia coli* as the most frequent pathogens, with *S. aureus* predominating in all geographical regions analysed [19]. Common resistant organisms include methicillin-resistant *S. aureus* (MRSA) and vancomycin-resistant *Enterococcus* [19,20,21]. Similar organisms are often responsible for burn infections, where the most prevalent pathogens are *Pseudomonas* spp., *Acinetobacter* spp. or *S. aureus* [22,23]. Important dermatological pathogens include *S. aureus*, *Propionibacterium* and *Streptococcus* spp. causing, among other pathologies, common skin infections such as acne, folliculitis, cellulitis and impetigo [24,25,26,27]. Other important, but less frequent, superficial bacterial pathogens include bacteria of the genera *Enterobacter*, *Proteus*, *Klebsiella*, *Aeromonas*, *Corynebacterium* and *Clostridium* [28,29].

The safety and efficacy of intravenous phage therapy has been reviewed elsewhere [30], however no such review exists for bacterial skin infections. Therefore, this systematic review aims to assess the safety and efficacy of phage therapy for superficial bacterial infections, specifically burn, chronic wound/ulcer or dermatological infections secondary to common causative organisms. This systematic review will examine reports of human clinical applications of phage therapy that meet these criteria, without limitation on study design or route of administration.

## 2. Methods

### 2.1. Search Strategy

Three electronic databases were searched, without limits, for articles published up to October 2019: EMBASE (1980–2019), Ovid MEDLINE^®^ Epub Ahead of Print, In-Process & Other Non-Indexed Citations, Ovid MEDLINE^®^ Daily, Ovid MEDLINE and Versions^®^ (1946–2019) and Web of Science. The Web of Science Core Collection Citation Indexes searched were: Science Citation Index Expanded (1900–2019), Book Citation Index—Science (2005–2019) and the Emerging Sources Citation Index (2015–2019). The search was performed using the following terms: (“Bacteriophage*” OR “Phage*”) AND (“Acinetobact*” OR “Aeromonas” OR “Clostrid*” OR “Corynebact*” OR “Diptheroid*” OR “Enterobact*”OR “Enterococ*”OR “Escherichia*” OR “Klebsiella” OR “Propionibacterium” OR “Proteus” OR “Pseudomonas” OR “Staphylococ*” OR “Streptococ*”) AND (“Acne” OR “Boil” OR “Burn*” OR “Carbuncle*” OR “Cellulitis” OR “Cutaneous abscess” OR “Derm*” OR “Dermatitis” OR “Eczema” OR “Erysipelas” OR “Folliculitis” OR “Furuncle*” OR “Hidradenitis” OR “Impetigo” OR “Infect*” OR “Pyoderma” OR “Skin” “Ulcer” OR “Wound”) AND (“Case*” OR “Clinic*” OR “Patient” OR “Treat*” OR “Trial”). In Ovid these terms were followed by the suffix ‘.mp.’ and they were searched as topics on the Web of Science platform. This systematic strategy was supplemented by hand searching of sources not widely available or indexed online [31,32,33], reports cited in review articles [34,35,36,37] or relevant papers that became available after the systematic search date [38]. A protocol was not published prior to this study.

### 2.2. Study Selection Criteria

All studies underwent title and abstract screening. Eligible studies met the following criteria: (A) they contained primary data pertaining to human clinical phage therapy by any route of administration for one or more superficial bacterial infections (burn, chronic wound/ulcer or dermatological infection) caused by bacteria of the genera *Staphylococcus*, *Pseudomonas*, *Streptococcus*, *Enterococcus*, *Escherichia, Aeromonas, Acinetobacter, Corynebacterium, Clostridium, Enterobacter, Proteus, Klebsiella* or *Propionibacterium*; (B) they were published in the English language. Chronic wounds/ulcers complicated by osteomyelitis were included, but cases of osteomyelitis secondary to other causes (e.g., trauma or surgery) were excluded. Studies in which the clinical condition was stated but the infectious agent was not specified were assumed to be caused by one of the major pathogens examined in this study and therefore included. There were only three such studies, none of which were considered to report conditions unlikely to be caused by the bacterial genera in the search strategy [31,39,40]. Secondary literature was excluded unless it reported primary clinical data unavailable from the primary source. However, secondary literature was preferentially included when it contained more primary clinical data than that available from a primary source alone. There were no limitations on study date, type or location. Studies that only used phage protein products (e.g., endolysins) were excluded. There was no limitation on the purity of phage preparation used and studies using raw bacterial lysate were included.

Deduplication was performed using Endnote (version X8.0.1). Title and abstract screening was performed independently by two authors (AS, SdS), with discrepancies resolved by a third author (JDJ). Full-text screening was performed independently by two authors (AS, JDJ), with discrepancies resolved by a third author (HJS). This review was conducted in accordance with the PRISMA (Preferred Reporting Items for Systematic Reviews and Meta-Analyses) guidelines [41] and a PRISMA checklist completed (Supplementary File S1).

### 2.3. Data Extraction & Critical Appraisal

The following information was extracted from each study: author(s); date of publication; study location; study type; number of relevant reports; condition microbiology; clinical condition, and where possible patient age(s) and/or previous treatment(s); details of the phage treatment; treatment schedule and route(s), including details of other ongoing therapies (e.g., antibiotics) where reported; treatment efficacy; where possible, the numbers of patients ‘cured’, ‘improved’ or for which there was ‘no response’; comments or data regarding safety and adverse effects. The term ‘cured’ is used in Supplementary Files 3–5 and is defined here as synonymous with clinical resolution of infection. For two papers that reported patients with recurrence of infection [42,43] these patients (*n* = 41) were added to the total patients with clinical resolution on the basis that recurrence, by definition, implies a period of clinical resolution. Slopek and colleagues reported three decubitus ulcer patients as having had ‘transient improvement’ [33]. These patients were deemed treatment failures as ‘transient’ improvement is a nebulous term that does not imply any period of resolution. Slopek and colleagues also reported seven patients with furuncles or skin inflammation as having ‘marked improvement’ with and negative bacterial culture, consequently these patients were classified as ‘improved’ by this review. Data extraction was performed independently by three authors (J.D.J., H.J.S. and A.S. or S.d.S.), with discrepancies resolved by agreement. All eligible studies were independently assessed by two authors (H.J.S and J.D.J) using the appropriate Joanna Briggs Institute critical appraisal checklist [44], with discrepancies resolved by agreement. The influence of publication bias and selective reporting on the cumulative evidence are considered in the discussion.

## 3. Results

**Figure 1:**
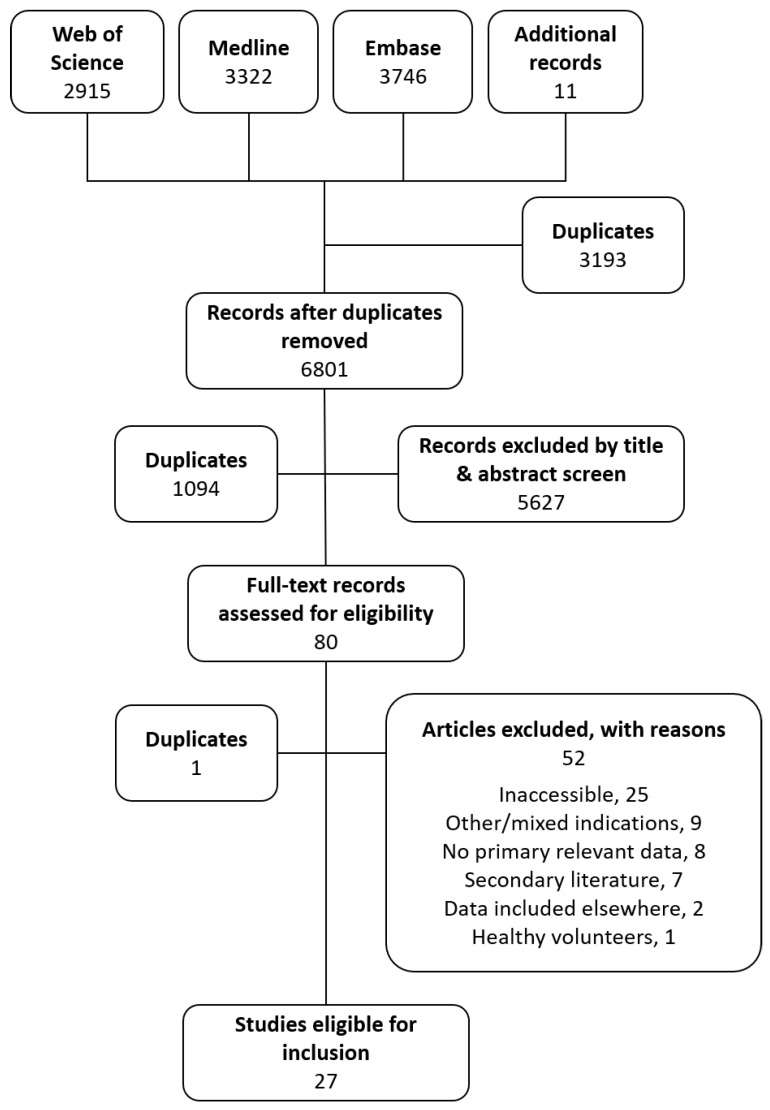
Flow diagram of study selection.

Systematic searching yielded 9983 records published between 1929 and 2019. Eleven additional records were identified from other sources; three from a review article [34], seven from grey literature sources known to the authors not to be indexed or available online [31,32,33] and another published after the search date but included for completeness [38]. Figure 1 illustrates the screening process.

A total of 27 eligible studies were identified for inclusion in this review [10,12,13,31,33,35,36,37,38,39,40,42,43,45,46,47,48,49,50,51,52,53,54,55,56,57,58]. The clinical cases documented in these studies were divided into three groups pertaining to the treatment of burn, chronic wound/ulcer or dermatological infection. Several manuscripts documented the treatment of one or more of these conditions and the data from such studies was divided among the three groups accordingly. Therefore, from a total of 27 studies, eight contained data on burns, 12 on chronic wounds/ulcers and 10 on dermatological infections. The 27 studies included manuscripts from US (*n* = 8), Georgia (*n* = 7), Poland (*n* = 2), Russia (*n* = 3), India (*n* = 2), Belgium (*n* = 1), Egypt (*n* = 1), France/Belgium (*n* = 1), Belarus (*n* = 1) and the UK (*n* = 1). There were two clinical trials, four case reports and 21 case series. Two of the 21 case series were reported as ‘prospective’ studies; however, it was deemed that these better suited established definitions of case series [38,58]. One study was originally reported as a clinical trial, but the nature of the intra-patient control used led to its classification as a case series for the purposes of this review [54]. Most (*n* = 24/27) studies reported microbiological data. This represented the application of phage therapy against nine of the 15 bacterial genera or species in the systematic search strategy (Table 1). Three studies did not include microbiological data but were discretionary inclusions as it was considered extremely likely that the clinical conditions reported would have been caused by one or more of the bacterial genera included in the search strategy. Critical assessment of the eligible papers highlighted an array of shortcomings in the quality of reporting, discussed later, but did not provide evidence of bias warranting exclusion (Supplementary File S2).

### 3.1. Bacteriophage Therapy for the Treatment of Burn Wound Infection

Eight records reported data regarding 156 patients with burn wound infections (Supplementary File S3), of which one was a phase I/II clinical trial, one a case report and six were case series. The reports were from Poland (*n* = 1), the US (*n* = 1), Belgium (*n* = 1), France and Belgium (*n* = 1), Georgia (*n* = 1), Russia (*n* = 1), Egypt (*n* = 1) and the United Kingdom (*n* = 1). Testing of the in vitro efficacy of phage against a patient’s bacterial isolate (phage sensitivity testing) was reported or implied for six of eight reports. Phage administration routes were oral (*n* = 1), topical (*n* = 6) or oral and local (*n* = 1). Topical administration included direct application (*n* = 3), the use of dressings soaked with phage solution (*n* = 2) or a biodegradable phage-containing bandage (*n* = 1). Prior neutralization of stomach acid was reported in one of two reports of oral phage administration. The phage treatments were unpurified phage lysates (*n* = 1), phage cocktails (*n* = 3), a phage-containing biodegradable bandage (*n* = 1) or unclear (*n* = 3).

A precise efficacy estimate cannot be derived, as co-administered therapies, phages and reporting timepoints and methodology differed between all records. Notwithstanding these caveats, and given that most infections reported were refractory to antibiotics, a crude and cautionary estimate of efficacy can be derived, partially excluding one report in which outcome data were unclear for 45/54 patients [37]. The remaining data represented 111 patients, of whom: 49.6% achieved clinical resolution, 27.9% showed improvement and 22.5% showed no improvement. Bacterial resistance to phage was recorded for three patients that did not reach the primary endpoint in the ‘Phagoburn’ clinical trial [12]. No other records commented on bacterial resistance to phage therapy.

Most (*n* = 5/8) studies did not comment on safety or adverse effects. Of the three studies that did, representing 20 patients, none reported adverse effects.

### 3.2. Bacteriophage Therapy for the Treatment of Infected Chronic Wounds or Ulcers

Twelve records reported data regarding 327 patients with chronic wound/ulcer infections (Supplementary File S4). There was one phase I safety trial, one case report and 10 case series. The reports were from the US (*n* = 5), Poland (*n* = 2), Russia (*n* = 2), India (*n* = 2) and Georgia (*n* = 1). The chronic wounds/ulcers treated were venous ulcers (*n* = 195; 59.6%), diabetic infections (*n* = 70; 21.4%), decubitus ulcers (*n* = 21; 6.4%), non-specific chronic non-healing wounds (*n* = 40; 12.2%) and community-acquired MRSA (*n* = 1; 0.3%). Phage sensitivity testing was reported or implied for five of 12 studies. The routes of phage administration were topical (*n* = 5), oral and local (*n* = 2), local injection/instillation (*n* = 2), local injection or topical (*n* = 1) or unclear (*n* = 2). Topical administration represented the use of dressings soaked with phage solution (*n* = 3), direct application (*n* = 1) or a biodegradable phage-containing bandage (*n* = 1). Where used, oral administration was preceded by neutralization of stomach acid. One report documented wound rinsing with 4% sodium bicarbonate prior to the application of a phage-containing bandage to venous stasis ulcers. The phage treatments used were unpurified phage lysate (*n* = 2), monovalent phage suspension (*n* = 3), phage cocktails (*n* = 2), monovalent or cocktail based (*n* = 2), a phage-containing biodegradable bandage (*n* = 1) or unclear (*n* = 2).

As for burn wound infections, a precise efficacy estimate could not be derived from these studies. Nevertheless, notwithstanding the aforementioned caveats, and given that most infections reported were refractory to antibiotics, a cautionary crude estimate of efficacy can be derived, excluding one report in which outcome data for all 17 patients was unclear [31]. The remaining 11 studies represented 310 patients, of whom: 65.8% reportedly achieved clinical resolution, 20.3% showed improvement and 13.9% showed no improvement.

Most (*n* = 7/12) studies did not comment on safety or adverse effects. Of the five that did, representing 141 patients, four reported no safety issues or adverse effects. Slopek and colleagues 1987 was the last in a series of papers, in which an earlier manuscript commented on the ‘rather rare’ adverse effects [59], including two instances of ‘oral intolerance’ to raw phage lysate and one of ‘allergic symptoms’ following local application were reported. Hepatalgia and fever were reported to have occurred several days after oral phage therapy and were described as ‘accounted for by the mass liberation of endotoxins from phage effect on bacteria’. The authors noted that phages are ‘safe’ and ‘present no danger’ to patients [59].

### 3.3. Bacteriophage Therapy for the Treatment of Dermatological Infection

Ten records reported data regarding 1096 patients with various bacterial skin infections (Supplementary File S5), of which eight were case series and two were case reports. Most reports were from Georgia (*n* = 5), with other reports from the US (*n* = 3), Poland (*n* = 1) and Belarus (*n* = 1). The infections treated were furunculosis (*n* = 606; 55.3%), hidradenitis (*n* = 94; 8.6%), acne (*n* = 68; 6.2%), impetigo (*n* = 67; 6.1%), carbunculosis (*n* = 64; 5.8%), ‘strepto-staphylococcal epidermitis’ (*n* = 25; 2.3%), abscesses (*n* = 14; 1.3%), sycosis vulgaris (*n* = 14; 1.3%), folliculitis (*n* = 11; 1.0%), dermatitis (*n* = 7; 0.6%), ecthyma (*n* = 8; 0.7%), cellulitis (*n* = 5; 0.5%), pyoderma (*n* = 4; 0.4%), paronychia (*n* = 1; 0.1%) and ‘various skin diseases’ (*n* = 102; 9.3%). Phage sensitivity testing was reported or implied for 4 of 10 studies. The routes of administration were oral and local (*n* = 2), subcutaneous or local (*n* = 1) or subcutaneous injection alone (*n* = 2) or supplemented by intranasal administration (*n* = 1) or local application (*n* = 4). Oral administration was preceded by neutralization of stomach acid in all cases. The phage treatments used were unpurified phage lysate (*n* = 3), monovalent and/or cocktail based (*n* = 2) or unclear (*n* = 5).

As before, a cautionary crude estimate of efficacy was derived, excluding two reports where outcome data for all 308 patients were unclear [39,46] and 1/57 and 53/143 patients reported by Larkum (1929) and Beridze and colleagues (1938) respectively for whom outcome data were incomplete. The remaining eight studies included represented 734 patients, of whom: 87.3% reportedly achieved clinical resolution, 6.81% showed improvement and 5.9% showed no improvement. One study commented on ‘resistance’ to phage therapy and the development of ‘antiphage’ [43]. Although neither term was defined in the study, the former was taken to mean the development of bacterial resistance to phage, of which there were nine reports, while the latter was interpreted as the action of anti-phage antibodies, occurring in one case.

Eight of 10 studies, representing 934 patients, commented on safety or adverse effects. Of these, one report from 2017 did not report any adverse effects or clinical or laboratory abnormalities. The remaining seven studies, dated 1929 to 1987, identified adverse effects. The earliest record, published 1929, reported that ‘extremely mild’ adverse effects were observed in the ‘majority’ of patients that received injected subcutaneous raw *Staphylococcal* phage lysate. Of the 149 patients for whom adverse effects were recorded, 28.8% had no adverse effects, 34.6% had mild localised erythema and soreness, 7.2% had generalized responses (fever, malaise), 1% had an undefined ‘severe’ reaction and no data were available for the remaining 28.4% [42]. One year later, Crutchfield and Stout reported a case series of 57 patients and noted a ‘severe local reaction’ for one patient; no other comments on safety were made and details of the phage product used were not reported [43]. The third record documenting adverse effects was published in Georgia in 1938 and reported localized and generalised adverse effects of subcutaneous phage injections, typically seen within, and lasting no longer than, 24–48 h. The authors suggested that the release of bacterial proteins because of phage-mediated lysis could trigger the patient’s immune system. This report did not contain any details about the nature of the phage preparation used [45]. Later Belarussian data from 1940 reported similar localized or generalized adverse effects [39]. Similar reactions to subcutaneous phage injections were again reported in Georgian data from 1937 and 1938–1941. Local reactions (e.g., ‘redness’, ‘inflammation’) were ‘often’ noted at the injection site, but this was ‘not an indication that phage treatment should be halted’. Additionally, an ‘allergic rash’ which ‘rapidly disappeared’ was observed in 8% of cases. The patients from this study were treated with staphylococcal or ‘pio’ bacteriophage preparations [46]. There were no details about the purity of the phage used in any of these Georgian or Belarussian studies. The sixth report of adverse effects came from a 1963 report of subcutaneous injection of raw phage lysate for furuncles [47]. ‘No systemic reactions’ were noted and ‘local reactions were mild and did not interfere with treatment’. Lastly, the comments of Slopek and colleagues 1987 were extracted from an earlier paper in the same series and are described above [59].

## 4. Discussion

This systematic review presents the available English language clinical data about the use of phage therapy for the treatment of superficial bacterial infections, specifically burn, chronic wound/ulcer or dermatological infection. A total of 27 relevant sources representing 1688 patients were identified, stretching back over 90 years. It is important to appreciate that this greatly underestimates the global extent to which phage therapy has been used. While phage therapy was disregarded by Western medicine, Russia and other Eastern European countries, particularly Georgia, continued to employ phage therapy [3], and today phage therapy products are still available over the counter in pharmacies [60]. Poland is also a major centre for phage therapy, with thousands of patients having been successfully treated at the Hirszfeld Institute of Immunology and Experimental Therapy [49]. There is therefore a great body of phage therapy literature that is unavailable in English and/or not indexed in electronic databases. Moreover, it is highly likely that, where phage therapy is frequently practiced, routine clinical applications go unreported. There are also early reports of Western phage therapy that are unavailable in English or not widely available [61]. Any systematic review of phage therapy performed in English, while still valuable, can therefore only offer a limited snapshot of the evidence supporting the safety and efficacy of phage therapy.

The studies identified in this review demonstrate that when used appropriately phage therapy should generally be considered highly effective. Although precise efficacy estimates could not be derived because no reports were directly comparable, cautionary crude efficacy estimates revealed that clinical resolution or improvement was achieved in most cases of chronic wound/ulcer treatment (86.1%, *n* = 310) and dermatological infection (94.9%, *n* = 734), although efficacy was not as high among burn wound infections (76.8%, *n* = 111). It should be noted that many of the patients in these studies were also receiving antibiotic therapy, although in most cases patients’ infections were deemed antibiotic resistant. The comparatively lower level of clinical resolution among burn wound infection patients (49.6%), reflects data from three studies. One study only reported degrees of improvement and did not document whether any patients had achieved clinical resolution (*n* = 30). This was despite reporting that post-phage therapy 60% of patients’ wounds were culture negative, wound discharge had stopped for 40% of patients and phage therapy had had a ‘good’ or ‘excellent’ effect on the take of skin grafts for 60% of patients [35]. Consequently, no cases from this report could justifiably be classified as having achieved clinical resolution. The two remaining studies were limited by technical challenges. In one, the titre of the phages used had dropped from the intended 10^6^ plaque forming units (PFU)/mL to just 10^2^ PFU/mL [12]. Although, because of phage replication, there is no classical dose-response curve with phage, the experience of topical phage therapy practitioners, reflected in this review, is that the therapeutic range for topical phage is generally between 10^6^ and 10^8^ PFU/mL. The other study was not, by the author’s admission, intended to evaluate efficacy and faced significant delays between patient identification and treatment [54]. The authors also suggested that efficacy may have been affected by their spraying of phage solution directly onto the wound, resulting in loss of phage solution as run-off. Excluding these three studies from the efficacy estimate for burn wound infections results in a revised estimate of 89.2% clinical resolution, rising to 100% when ‘improved’ patients are included (*n* = 65).

Unlike antibiotic therapy, the success of phage therapy is highly dependent upon the phage and bacterial species or strain in question, reflecting the specificity of phage/host interactions. It is believed that, unlike antibiotics, phage therapy therefore leaves commensal flora largely unaffected [62]. However, off-target effects may occur as a result of the use of phages with a broad host range or as a consequence of phage evolution during treatment, the impact of such effects is not well understood, but it is likely to be significantly less than the effects of antibiotics on commensal flora. In general, the specificity of phages means that careful matching of a phage to a patient’s bacterial isolate is required. However, the results of phage sensitivity testing were not generally reported. Where sensitivity data were available, it was unclear if it had been pro- or retrospectively obtained and it generally seemed that patients with resistant isolates were treated nonetheless. This may reflect that, despite a strong correlation between in vitro and in vivo efficacy [63,64], negative in vitro susceptibility does not always preclude in vivo efficacy [45]. Likewise, in vitro susceptibility, although a useful indicator, is not a guarantee of in vivo efficacy, which may be influenced by other host factors [64]. Nevertheless, an absence of phage susceptibility testing likely contributed to the numbers of patients that did not improve with phage therapy. Notably, the injudicious use of phage by enthusiastic Western clinicians in the 1920s and 1930s, yielding no or poor effects, contributed to the Western demise of phage therapy. The efficacy of phage therapy is also affected by the bacterial diversity of the infection. Seven reports stated that a proportion of the infections treated had multiple bacterial species or strains [12,33,38,45,50,55,65]. Six of these seven studies had patients who only either improved or did not respond to therapy, suggesting that the ‘primary’ pathogen may not have been effectively targeted or that, if removed by phage, it was replaced by other unaffected or resistant organisms.

Several manuscripts reported notable levels of treatment failure. A case series of burn wound infection patients noted that the development of resistance can present an obstacle when targeting *Pseudomonas* species, although whether the authors encountered resistance is unclear [35]. Bacterial resistance to phage was reported in three other studies included in this review [12,43,56]. Nevertheless, such is the diversity of phage that upon encountering resistance a suitable alternative phage can often be identified and substituted [6,56]. Moreover, the use of phage cocktails is an essential strategy to minimizing the development of bacterial resistance [14]. Although not bacterial resistance, one report described the development of ‘antiphage’, interpreted as anti-phage antibodies [43]. Antibodies can be raised against phage, although no adverse effects have been linked to antibodies and more recent data has questioned their impact on the efficacy on phage therapy [66,67]. The efficacy of phage therapy in cases of acne was particularly poor [42,43]. The partial efficacy of phage against *S. aureus* in cases of acne identified by this review likely reflects that acne has a complex pathophysiology [68]. A range of bacteria may be associated with acne [69], typically *Propionibacterium acnes*, although only phage therapy against *S. aureus* was identified in this review. *S. aureus* can survive antibiotic therapy in an intracellular non-dividing persister phenotype [70]. It is possible that the intracellular state of such cells may impart resistance to phage therapy and lead to treatment failure or clinical recurrence. However, phages against other bacteria associated with acne have been identified [11,71,72]. Lastly, a phase I safety trial in which 18 patients had a phage cocktail applied to chronic venous leg ulcers, was explicitly not a trial for efficacy and did not employ phage sensitivity screening or contain any investigation of patient’s clinical microbiology [53].

Surprisingly, 12 of the 27 studies identified by this review did not comment on safety or adverse effects. This likely reflects the inherent tendency of scientific reports to only document positive findings, with the authors not deeming the absence of adverse effects remarkable. As phage therapy seeks to regain traction it will be vital that all future reports comment on adverse effects, even if absent. Of the 15 studies that commented on adverse effects, none of the eight studies published after 2002 reported adverse effects (*n* = 146), including a US Food and Drug Administration approved phase I safety trial. This is consistent with the absence of adverse effects from other recent applications of phage therapy for other indications, including intravenous use [9], and likely reflects the use of purer phage preparations. A 2016 report investigated the antibody responses of patients and healthy volunteers to phage therapy and did not identify any adverse effects, even when notable levels of anti-phage antibodies were elicited [66]. This report did not meet the strict inclusion criteria for this review because it evaluated the effect of phage on healthy human volunteers or conditions beyond the scope of this review. The seven reports that identified adverse effects were dated 1929–1987: Six reported subcutaneous injection of phage for dermatological infections, supplemented by local application in some cases, and one the use of oral and local phage lysates for a variety of indications. Of the six papers reporting subcutaneous administration, two used raw phage lysate [42,47], one used a phage cocktail but did not comment on purity [46] and there were no details of the phage used by the three other reports [39,43,45], although given their dates, it is highly likely that raw phage lysates were also used. Slopek and colleagues’ report of oral intolerance reflects the administration of raw phage lysate [59], likely still in bacterial culture broth. Their report of ‘allergic symptoms’ in a case of local application may reflect a local response to endotoxin or other contaminants. While allergic responses to phage cannot be theoretically excluded, we are not aware of any confirmed reports of allergy to phage. Impure phage preparations contain high concentrations of lysed bacteria that would inevitably be recognised by the immune system and trigger localised or general acute inflammatory responses, as may be observed for vaccines [73]. The authors of two studies suggested that the lytic action of phage might contribute to the release of inflammatory bacterial proteins, provoking an immune response [45,65]. This has been a concern with phage therapy against some bacteria, particularly endotoxin from Gram-negative bacteria [3]. However, recent evidence suggests that in some cases phage may have a lower endotoxin release profile than some antibiotics [74]. Nevertheless, it is reassuring that the studies identified here, which represent a broad range of phage/bacteria combinations, do not report any adverse effects that cannot be readily explained by the mode of administration (e.g., injection) or the presence of bacterial contaminants. The negligible side effect profile of phage therapy identified by the studies included in this review illustrates how advantageous phage therapy is relative to antibiotic therapy, which often causes adverse effects. However, it is important to note that the safety of every different phage used for therapy must be assessed. Extensive literature about the characterisation of phage for therapy is available elsewhere [75,76,77], with primary concerns being the exclusion of phage with lysogenic, toxin or antimicrobial resistance genes.

The reliance on English language sources available on the internet is an insurmountable limitation of this review. Inaccessibility was the most common reason that manuscripts were excluded during the full text screen, typically reflecting manuscripts published in volumes that were unavailable online or concealed by a paywall. Had all 26 such manuscripts been available in full, nine would have only been available in a foreign language. It was for this reason that the authors sought to supplement the data included in this review by including primary clinical data from secondary sources. This reflects the unique importance for any English language literature review of phage therapy to engage with less accessible sources. 

As with any systematic review, the quality of the data extracted is dependent upon the quality of primary reporting. Critical appraisal of the manuscripts included in this review revealed a variety of shortcomings, from not commenting on the presence or absence of adverse effects to inconsistencies with patient numbers reported or a lack of clarity about the condition or clinical microbiology of patients. Systematic reviews are also inherently limited by whether relevant studies contain key search terms in the fields searched and are available or appropriately indexed in the databases used. As renewed interest in phage therapy grows, it is important that all reports of phage therapy present their findings clearly and fully, especially regarding adverse effects and that, where possible, reports are published in indexed open-access journals.

## 5. Conclusions

In summary, this review has collated English-language evidence to support the view that phage therapy for superficial bacterial infections should generally be considered both safe and effective. We hope this will encourage the development of complimentary reviews of phage data currently not available in English. Phage therapy for such infections has a long history and offers a potentially cheap, easy-to-use adjunct or alternative to antibiotics. Aside from reducing the morbidity and mortality of these infections, the use of phage also has the potential to reduce the amount of antibiotics prescribed for such infections, some of which often require multiple courses of antibiotics. Moreover, with the potential to generate bespoke formulations, phage therapy can offer, by definition, ‘personalised infection medicine’.

## 6. Declarations

### Ethics Approval and Consent to Participate

This article does not contain any studies with human participants or animals performed by any of the authors.

## Figures and Tables

**Table 1:** Bacterial genera and species for which reports of phage therapy were identified by the systematic search strategy.

	Bacterial Genera and Species Targeted by Phage Therapy			
	Chronic Wound/Ulcer Infections	Burn Wound Infections	Dermatological Infections	No Reports
*Acinetobacter*				✓
*Aeromonas*				✓
*Clostridium*				✓
*Corynebacterium*				✓
*E. coli*	✓	✓	✓	
*Enterobacter*				✓
*Enterococcus*	✓	✓		
*Klebsiella*	✓	✓		
*P. aeruginosa*	✓	✓	✓	
*Propionibacterium*				✓
*Proteus*	✓	✓		
*S. aureus*	✓	✓	✓	
*S. epidermidis*	✓			
*S. lugdenensis*	✓			
*Streptococcus*	✓		✓	

## Supplementary Materials

The following are available online at https://www.mdpi.com/2079-6382/9/11/754/s1, Supplementary File S1: PRISMA checklist, Supplementary File S2: Critical appraisal, Supplementary File S3: Phage therapy for the treatment of burn wound infection, *n* = 9, Supplementary file S4: Phage therapy for the treatment of chronic wound or ulcer infections, *n* = 12, Supplementary file S5: Phage therapy for the treatment of dermatological infections, *n* = 11.

## Abbreviations

MRSA	methicillin-resistant *Staphylococcus aureus*
MSSA	methicillin-susceptible *Staphylococcus aureus*
PFU	plaque forming unit
PRISMA	Preferred Reporting Items for Systematic Reviews and Meta-Analyses
SPL	*Staphylococcus* phage lysate
