# Supplementary material for: The Safety and Efficacy of Phage Therapy for Superficial Bacterial Infections: A Systematic Review
Source: Antibiotics (Basel). 2020 Oct 29;9(11):754. doi: 10.3390/antibiotics9110754 (PMC7692203; doi:10.3390/antibiotics9110754)
Supplement: Supplementary file 1 [file antibiotics-09-00754-s001.zip › Supplementary file 2.pdf]

Supplementary file two: critical appraisal

Critical appraisal of manuscripts was performed using the Joanna Briggs Institute suite of critical appraisal tools available from https://joannabriggs.org/critical-appraisal-tools [accessed 14/09/2020]. The case series tool was adapted to include comments on adverse effects and, where appropriate, examples of what each question was interrogating.

Case series

Author (year), [citation]	Clear inclusion criteria?	Reliable standard measurement of presenting condition?	Valid identification of infection (e.g. defined microbiology)?	Consecutive inclusion?	Complete inclusion and reporting?	Clear patient demographic data (e.g. range and mean of patient age)?	Clear clinical reporting (pre- treatment)?	Clear outcome or follow up reporting (e.g. clinical outcome)?	Comments on adverse effects?	Clear site demographic (e.g. study location)?	Appropriate statistical analysis, if present?	Comments
Larkum (1929), [40]	No	Unclear	No	Unclear	Yes*	No	Yes	Yes	Yes	Yes	N/A	*Numerical discrepancy of one between raw and summary data. Raw data used.
Crutchfield & Stout (1930), [41]	No	Unclear	No	Unclear	No*	Yes	Yes	Yes	Yes	No	N/A	*States 119 patients treated, but only 57 reported.
Beridze *et al.*(1938), [43]	No	Unclear	Unclear	Unclear	Yes	No	Yes	Unclear*	Yes	Yes	N/A	*Outcome data not fully reported for impetigo and 'various skin diseases'. Numerical discrepancy of one between raw and summary data. Raw data used.

Bernstein *et al.* (1940), [58]	No	Unclear	No	Unclear	Yes*	No	No	No	Yes	Yes	N/A	*Yes for hidradenitis (22/141). Data for furunculosis (119/141) contained irreconcilable numerical discrepancies and was consequently excluded.
Vartepetov (1957), [44]	No	Unclear	Unclear	Unclear	Yes	No	No	No	Yes	No	N/A	
Baker (1963), [45]	No	Unclear	No	Unclear	Yes	No	Yes	Yes	Yes	Yes	N/A	
Shvelidze (1970), [46]	No	Yes	Yes	Unclear	Yes	No	Yes	Yes	No	No	N/A	
Slopek *et al.* (1987), [33]	No	Yes	Yes	Unclear	Yes	No	Yes	Yes	Yes*	Yes	N/A	*See preliminary paper Slopek *et al.* 1983 [59]
Abul-Hassan *et al.*1990, [35]	Yes	Yes	Yes	Unclear	Yes	No	Yes	Yes	No	No	N/A	
Weber-Dabrowska, Mulczyk & Górski (2000), [47]	No	Yes	Yes	Unclear	Yes	No	No	Yes	No	Yes	N/A	
Lazareva *et al.* (2001), [37]	No	Unclear	Yes	Unclear	Yes	No	No	Unclear	No	Unclear	N/A	
Markoishvili *et al.* (2002), [48]	No	Yes	Unclear*	Yes	Yes	No	Unclear**	Yes	Yes	Yes	N/A	*Microbiological data presented for 22/96 patients. **Clear data given for 22/96 patients.
Jikia *et al.* (2005), [49]	No	Yes	Yes	Yes	Yes	Yes	Yes	Yes	No	Yes	N/A	
Southwest Regional Wound Care Centre (2006), [31]	No	Yes	No	No	Unclear	Yes	Yes	Yes	No	Yes	N/A	

Rose *et al.* (2014), [52]	Yes	Yes	Yes	Unclear	Yes	No	Yes	Yes	Yes	Yes	N/A	
Fish *et al. (*2016), [10]	Yes	Yes	Yes	Unclear	No*	Yes	Yes	Yes	Yes	No	N/A	*Nine patients treated; six 'representative' cases presented.
Vlassov *et al.* (2016), [36]	No	Unclear	Yes	Unclear	Yes	No	No	Unclear	No	No	N/A	
Fish *et al. (*2018), [53]	Yes	Yes	Yes	Unclear	Yes	Yes	Yes	Yes	Yes	No	N/A	
Morozova *et al.* (2018), [55]	No	Yes	Unclear	No	Yes	No	Unclear	Yes	No	No	N/A	.
Gupta *et al.*2019, [56]	Yes	Yes	Yes	Unclear	Yes	No	Yes	Yes	No	Yes	Yes	
Patel *et al.*2019, [38]	Yes	Yes	Yes	Unclear	Yes	Yes	Yes	Yes	No	Yes	Yes	

Case reports

Author (year)	Were the patient's demographics clearly reported?	Was the patient's history clearly described?	Was the current clinical condition clearly described?	Were diagnostic tests or methods and results clearly described?	Were the treatment(s) or intervention(s) clearly described?	Was the post-intervention clinical condition clearly described?	Were any adverse or unanticipated events clearly described?
Izashvili (1957), [57]	Yes	Yes	Yes	Yes	Unclear	Yes	Unclear
Marza *et al.* (2006), [50]	Yes	Yes	Yes	Yes	Yes	Yes	Yes
Zhvania *et al.* (2017), [54]	Yes	Yes	Yes	Yes	Yes	Yes	Yes
Fish *et al.* (2018), [13]	Yes	Yes	Yes	Yes	Yes	Yes	Yes

Clinical trials

Author (year)	True randomisation?	Allocation to treatment groups concealed?	Treatment groups similar at baseline?	Participants blind to assignment?	Treatment givers blind to assignment?	Outcome assessors blind to assignment?	Treatment groups treated identically - other than the intervention	Complete follow up?	Participants analysed in allocated groups?	Consistent outcome measurement?	Reliable outcome measurement?	Appropriate statistical analysis?	Was the trial design appropriate?
Rhoads *et al.* (2009), [51]	Yes	Yes	Yes	Yes	Unclear	Unclear	Yes	Yes	Yes	Yes	Yes	Yes	Yes
Jault *et al.* (2019), [12]	Yes	Yes	Yes	Yes	No	Yes	Yes	Yes	Yes	Yes	Yes	Yes	Yes
