# Supplementary material for: The Safety and Efficacy of Phage Therapy for Superficial Bacterial Infections: A Systematic Review
Source: Antibiotics (Basel). 2020 Oct 29;9(11):754. doi: 10.3390/antibiotics9110754 (PMC7692203; doi:10.3390/antibiotics9110754)
Supplement: Supplementary file 1 [file antibiotics-09-00754-s001.zip › Supplementary file 3.pdf]

Supplementary file three: phage therapy for the treatment of burn wound infections

Report details		Clinical details				Efficacy				Safety & adverse effects
**Author (year), [citation], location, study type**	**No. of relevant reports and microbiology**	**Condition details**	**Phage sensitivity**	**Phage treatment**	**Treatment schedule and route(s)**	**Outcome**	**Cured**	**Improved**	**No response**	
Abul-Hassan *et al.* (1990), [35], cited in [34]<br><br>Egypt<br><br>Case series	30/30<br><br>*Pseudomonas* infection of burn wounds, resistant to all available antibiotics and chemotherapeutic agents.	6/30 patients were children aged <9, the rest were aged 18-54. Burn wound infection area ranged from 10-21% of body surface area.	Sensitivity confirmed.	*Pseudomonas* phage at 10^{10}PFU/ml. No further details.	Gauze soaked in phage was applied 3 times daily for 5-17 days as required. Use of concurrent antibiotic therapy unclear.	After phage therapy, 12/30 had negative microbiology; 15/30 had improved, 9/30 had slight improvement and 6/30 had no improvement; discharge stopped in 12/30, diminished in 12/30 and didn't change in 6/30; the effect of phage therapy on skin graft taking was excellent in 6/30, good in 12/30 and poor in 12/30.	0	24	6	No comment.
Weber-Dabrowska, Mulczyk & Górski (2000), [47]<br><br>Poland<br><br>Case series	49/1307<br><br>Infected with *S. aureus, E. coli, Klebsiella, Proteus* or *Pseudomonas.*	Pyogenic burns. Most were chronic infections that had failed antibiotic therapy.	Combined sensitivity data presented for all 1307 patients.	Crude, sterile, bacteriophage lysate.	Three times daily. Adults 10ml, children 5ml.<br><br>Orally and '30 minutes before eating, after neutralisation of the gastric juice'<br><br>Local administration also used on a case-by-case basis, details unclear.<br><br>Therapy duration for all 1307 patients reported as ranging from 1-12 weeks, with an average of 32 days.	42/49 recovered fully.<br><br>7/49 had 'marked improvement' but bacteria were still detectable.	42	7	0	No comment.
Lazareva *et al.* (2001), [37], cited in [34]	54/54<br><br>Organisms: *Enterococci*, *E. coli*, *P. aeruginosa*	Burn wound infection.	No phage sensitivity testing reported.	'Pyophage' cocktail.	Oral therapy. '2 tablets 3 times a day in 1-1.5h before meals' for 7 days. 45 patients treated with antibiotics and phage. Nine treated with phage only.	For patients treated with antibiotics and phage the number of microbial isolates in wounds dropped by 2.2x and positive blood culture	Data unclear.			No comment.

Russia<br><br>Case series	and *Staphylococci*.					results dropped 55 to 37%. Wound healing in 9/9 patients treated with phage only.	9	0	0	
Jikia *et al.* (2005), [49]<br><br>Georgia<br><br>Case series	2/3<br><br>*S. aureus* isolated from burns.	Two males aged 45 and 52 with radiation burns infected with *S. aureus*<br><br>Previous treatment with intravenous antibiotics and antimicrobial ointments for 23 days was unable to resolve the infections.	Sensitivity confirmed.	'Phagobioderm'. A biodegradable bandage containing a mix of chemotherapeutics and phages:<br><br>10^6 PFU/ml 'Pyo' phage cocktail (vs. *S. aureus, P. aeruginosa, E. coli, Streptococcus* and *Proteus*)<br><br>Also: ciprofloxacin (0.6mg/cm^2); benzocaine (0.9mg/cm^2); α-chymotrypsin (0.05mg/cm^2); sodium bicarbonate (3.75mg/cm^2)	A single application of 'Phagobioderm' immobilised with sterile bandages<br><br>Wounds examined daily for 5 days and once every 2-4 days thereafter. Microbiology monitored, frequency not reported.	Two days after application of Phagobioderm purulent discharge from both patients reduced to 'almost none'.<br><br>On day 7 both ulcers tested negative for *S. aureus* and both were able to receive skin grafts.<br><br>The *S. aureus* isolates from both patients were later shown to be resistant to ciprofloxacin.	2	0	0	No comment.
Marza, Soothill, Boydell & Collyns (2006), [50]<br><br>UK<br><br>Case report	1/2<br><br>*P. aeruginosa* infection.	50% surface area burns with skin grafts applied. Refractory to appropriate antibiotic treatment.	Sensitivity confirmed.	Pseudomonas phage at 10^{15} PFU/ml.	10^3 PFU of phage in 0.2ml was absorbed into two 25mm sterile filter paper discs which were placed on the infected areas.<br><br>After 48h the discs contained 4.3 x 10^4 and 1.2 x 10^6 PFU respectively, showing phage replication.	*Pseudomonas* was not isolated from the wound 3 days after treatment and subsequent extensive skin grafting was successful.	1	0	0	'No toxicity to human keratinocytes was observed when they were cultured with the purified phage suspension.'<br><br>'Several hours [after application to the whole wound] the patient had a

					Phage was then applied to the whole wound area.<br><br>Phage was used alongside IV ceftazidime.					febrile episode, but similar episodes had occurred previously and otherwise no adverse effects were observed.’
Southwest Regional Wound Care Centre, 2006, [31]<br><br>US<br><br>Case series	1/28<br><br>MRSA positive infected burn wound.	MRSA positive burn wound of approx. 2 months duration in a 60-year-old male on methotrexate and high-dose prednisone for autoimmunity.	No phage sensitivity testing reported.	No details.	Wound debrided and phages applied weekly as part of wound care.	Complete healing in 11 weeks.	1	0	0	No comment.
Rose *et al.* (2014), [52]<br><br>Belgium<br><br>Case series	9/9<br><br>Multi-drug resistant *P. aeruginosa* and/or *S. aureus* infection.	Burn-wound infection. One patient had two burn wound infections.<br><br>Mean patient age was 61 years old (range, 27-88 years old). Mean total burned surface area was 30% (range, 6-45%).	Sensitivity confirmed.	‘BFC-1’ cocktail, containing lytic phages active against *P. aeruginosa* (14/1 and PNM) and *S. aureus* (ISP), each at 10^9 PFU/ml.	BFC-1 was applied in a single topical dose at an average of 0.03ml per cm^2.<br><br>The cocktail was applied to half of a patient’s wound area, the other half of the wound received standard care. Punch biopsies of each half of the wounds were taken before and 2-4h after BFC-1 therapy. Five phage applications took place during surgery, prior to which wounds had been cleansed with 5% Hibitane and filtered water. Average area of phage administration was $95cm^2$ (range, 25-$150cm^2$).<br><br>Patients with *P. aeruginosa* were also treated with one dose (25mg/kg initially) of amikacin in combination with ceftazidime (1g initially) or meropenem (2g/8h). Patients with *S. aureus* infection were also treated with vancomycin (1g initially) or linezolid (1200mg/day).	The bacterial loads were found to be very low in 8/10 wounds, owing to delays in admission to the trial during which patients received intensive antibiotic therapy.<br><br>In all cases, the biopsy bacterial load was unchanged after phage application.<br><br>The authors note that they did ‘not [expect] that a one-off application […] on a small wound surface would generate conclusive proof of the efficacy’.	0	0	9	‘No adverse effects, clinical abnormalities or changes in laboratory results that could be related to the application of phages were observed.’

Supplementary file three: phage therapy for the treatment of burn wound infections

Jault *et al.* (2019), [12]<br><br>France & Belgium<br><br>Clinical trial	10/12<br><br>Two did not receive a full course.<br><br>*P. aeruginosa* infected burns.	Average patient age was 61, with an average of 19% of body surface area burned, with an average of 6% third degree burns. Two patients were receiving antibiotic therapy at the time on enrolment.	Sensitivity confirmed.<br><br>Amongst those who did not reach the primary endpoint 3/10 had resistant colonies.	PP1131 cocktail (12 lytic phages)<br><br>Planned 10^6 PFU/ml, but deteriorated to 10^2 PFU/ml (considered subtherapeutic).	Once daily topical application for 7 days. Adjunctive antibiotic therapy at the discretion of the treating physician according to French Burn society recommendations.	Primary outcome was time taken for a sustained reduction in bacterial burden of two quadrants or more assessed by semi-quantitative culture results:<br><br>Phage group: median 144h (95% CI, 48h to not reached)<br><br>Control group: 47h (95% CI, 23-122h)	0	0	10	'Safety assessments included frequency, duration and severity of adverse events, clinical laboratory tests, vital sign measurements, physical examinations and clinical assessment of burn wounds.'<br><br>'Adverse events' reported among 23% of phage patients and 54% of control patients. No substantial difference between the groups.<br><br>Although the phage titre was 10^2 PFU/ml, the equivalent of 10^6 phage particles (most inactive) were applied to patients and 'did not provoke safety issues'.
