# Supplementary material for: The Safety and Efficacy of Phage Therapy for Superficial Bacterial Infections: A Systematic Review
Source: Antibiotics (Basel). 2020 Oct 29;9(11):754. doi: 10.3390/antibiotics9110754 (PMC7692203; doi:10.3390/antibiotics9110754)
Supplement: Supplementary file 1 [file antibiotics-09-00754-s001.zip › Supplementary file 4.pdf]

Supplementary file four: phage therapy for the treatment of chronic wound or ulcer infections

Report details		Clinical details					Efficacy				Safety & adverse effects
Author (year), [citation], location, study type	No. of relevant reports and microbiology	Condition details		Phage sensitivity	Phage treatment	Treatment schedule and route(s)	Outcome	Cured	Improved	No response	
Slopek *et al.* (1987) [33]<br><br>Poland<br><br>Case series	16/550<br><br>Staphylococci in all cases. 14/16 were polymicrobial.	Decubitus ulcer	Antibiotic resistant.	No phage sensitivity testing reported. However, according to (Slopek *et al.*1983 [59]), sensitivity confirmed, results not shown.	No details. However, according to (Slopek *et al.*1983 [59]), a library of 259 bacteriophages was available for use. Crude phage lysates were used therapeutically.	Oral and local phage therapy was used.	'Favourable effect' in 13/16. Ineffective in 3/16.	13	0	3	No comments specific to these patients.<br><br>However, according to (Slopek *et al.* 1983 [59]): 'side effects in the course of phage therapy are very rare. Out of 138 [patients] only 3 cases were recorded of which 2 displayed drug intolerance at oral administration and 1 allergic symptoms at local application on the wound.'<br><br>'On day 3-5 of phage therapy, hepatalgia occurred which lasted several hours. This can be accounted for mass liberation of endotoxins

											resulting from phage effect on bacteria. [In] severe cases with sepsis, an increase of temperature occurred on day 7 - 8 of phage administration which lasted 24 h.'<br><br>'Bacteriophages are safe, side effects are rather rare and present no danger for a patient, they are transient and easy for restraint.'
Weber-Dabrowska, Mulczyk & Górski (2000), [47]<br><br>Poland<br><br>Case series	77/1307<br><br>Infected by *S. aureus, E. coli, Klebsiella, Proteus* or *Pseudomonas.*	Venous ulcer	Mostly chronic infections that had failed antibiotics.	Combined sensitivity data presented for all 1307 patients.	Crude, sterile, bacteriophage lysate.	Three times daily. Adults 10ml, children 5ml.<br><br>Orally and '30 minutes before eating, after neutralisation of the gastric juice'.<br><br>Local administration also used on a case-by-case basis, 'depending upon localisation of the supparative process'.<br><br>Therapy duration for all 1307 patients reported as ranging from 1-12 weeks, with an average of 32 days.	47/77 recovered fully.<br><br>21/77 showed 'marked improvement', but bacteria were still detectable.<br><br>9/77 no effect.	47	21	9	No comment.
Markoishvili *et al.* (2002), [48]<br><br>Georgia	96/96<br><br>Infected by *S. aureus, S. epidermidis, P.*	Venous ulcer	Venous stasis ulcers clinically judged to be refractory to standard	Sensitivity confirmed.	'PhagoBioDerm'. A biodegradable bandage containing a mix of chemotherapeutics and phages:	Wounds rinsed with sterile 4% sodium bicarbonate prior to the application of PhagoBioDerm.	67/96 achieved complete healing, with recovery ranging from 6 days to 15 months.	67	24	5	'No systemic effects of therapy were observed in any of the patients'

Case series	*aeruginosa, E. coli* or β-haemolytic *streptococcus*. 21/22 cases where microbiological data were available were polymicrobial.		therapy. Patients aged 31-101 years old.		10^6 PFU/ml 'Pyo' phage cocktail (vs. *S. aureus, P. aeruginosa, E. coli, Streptococcus* and *Proteus*)<br><br>Also: ciprofloxacin (0.6mg/cm^2); benzocaine (0.9mg/cm^2); α-chymotrypsin (0.05mg/cm^2); sodium bicarbonate (3.75mg/cm^2).	Wounds were examined daily for the first 5 days and once every 2-4 days thereafter. Microbiological monitoring data were available for 22 patients.<br><br>PhagoBioDerm was replaced when fragmented or degraded, typically every 3-7 days, or left in place if tightly attached or the wound had healed.<br><br>PhagoBioDerm was used as part of treatment schedules that varied between patients, some of which included clindamycin, Daflon or metronidazole.	Reduction in ulcer size and discharge was observed in 24 patients.<br><br>In 5 patients no improvement was seen. All had poorly controlled diabetes, one stopped phage after 1 week, the other four stopped after 1 month.<br><br>All 22 patients with microbiological monitoring showed bacterial counts eliminated or reduced by an average of 100-fold.				
Southwest Regional Wound Care Centre, 2006, [31]<br><br>US<br><br>Case series	17/28<br><br>Few microbiological details, presumed common skin pathogens, phages used against *P. aeruginosa* in one case.	Venous ulcer (n = 4)<br>Diabetic infection (foot; n = 7)<br>Decubitus ulcer (n = 5)<br>Community-acquired MRSA (CA-MRSA; n = 1)	Patients aged 53-87<br>Patients aged 45-73<br>Patients aged 36-93<br>Patients aged 34	No phage sensitivity testing reported.	No details for most cases. Phages against *P. aeruginosa* in one case.	No details of phage therapy provided. Phage was used as part of a varied package of care including debridement, wound care, 'biofilm management' and hyperbaric oxygen in some cases.	Improvement reported in all patients. Outcome data inconsistently recorded. 2/4 venous ulcers healed in 8 and 12 weeks. 2/7 diabetic foot infections healed in 3.5 and 10 weeks. 1/4 decubitus ulcers healed in 4 weeks.1/1 CA-MRSA healed in 35 days.	Data unclear.			No comment.
Rhoads *et al.* (2009), [51]	18/20<br><br>Presumed *S. aureus, P.*	Venous ulcer	Two patients in the treatment group (n = 20) dropped out for	No phage sensitivity testing reported.	Phage cocktail WPP-201.	4ml of WPP-201 in 46ml of saline. Instilled once weekly for 12 weeks by ultrasonic debridement machine at a drip rate of 200ml/h.	Outcome was assessed at 12 weeks based on degree of closure with follow up at 16 and 24 weeks.	0	0	18	'No adverse effects were attributed to the study product'. No

Supplementary file four: phage therapy for the treatment of chronic wound or ulcer infections

US<br><br>Clinical trial	*aeruginosa* and *E. coli.*		unknown reasons, although no adverse effects were observed or reported. Patient mean age was 62.8.		Contains eight types of phage active against *S. aureus, P. aeruginosa* and *E. coli*, each at 10^9 PFU/ml and suspended in phosphate-buffered saline.	Dressings (Promogran, Acticoat, Allevyn and three-layer compression bandages) were applied with Bovine lactoferrin (1%) and xylitol (5%) topical gel.<br><br>Antibiotic administration was permitted where signs of acute inflammation were observed.	No significant difference in wound healing was observed between the control (n = 19) and treatment (n = 18) groups.<br><br>The study number was directed by the FDA and the trial was designed as an efficacy study.				significant differences in the quantity or quality of adverse effects was observed between the two groups.
Fish *et al.* (2016), [10]<br><br>US<br><br>Case series	9/9<br><br>Infected with MRSA (n = 1) or MSSA (n = 8).	Diabetic infection (toe)	Detailed case report information provided for 6/9 patients aged 44-74. In 5/6 reports the patients had failed one or more courses of antibiotics, 1/6 patients were treated prophylactically. All patients had vascular insufficiency.	No phage sensitivity testing reported.	Monovalent suspension of anti-Staphylococcal phage Sb-1 at ~10^7-10^8 PFU/ml.	Once weekly applications of 0.1-0.5cc. Phage was dripped into the wound cavity, which was packed with phage-soaked gauze, covered with petroleum gauze and dry gauze. The phage dressing was left in place for 48h. Phage therapy supplemented standard wound care.	All infections responded and healed in an average of 7 weeks. The patient treated prophylactically healed without infection.	9	0	0	'No observed adverse effects during treatment' and 'no tissue breakdown or recurrence'.

Supplementary file four: phage therapy for the treatment of chronic wound or ulcer infections

Vlassov *et al.* (2016), [36], cited in [34]<br><br>Russia<br><br>Case series	23/23<br><br>Pathogens: *E. coli, Klebsiella* spp., *P. aeruginosa, Proteus* spp. and *Staphylococci*.	Diabetic infection (foot)	-	No phage sensitivity testing reported.	Phages against: *Staphylococci*, *Pseudomonas, E. coli* and *Enterococcus* at 10^8-10^{10} PFU/ml in combination with antibiotics.	Wound washing and topical application 1-4x daily for 5-14 days.	Elimination of *S. aureus* or *E. coli. or P. aeruginosa* titre fell 3-4 orders in 13/13 patients with monomicrobial infections. Elimination or decrease in bacterial titre in 4/10 patients with polymicrobial infections.	17	0	6	No comment.
Morozova *et al.* (2018), [55]<br><br>Russia<br><br>Case series	2/2<br><br>Pathogen: MRSA.	Diabetic infection (foot, hand)	-	No phage sensitivity testing reported.	Different therapies in each case: 'Piobacteriophage' (Microgen) or 'lytic *Staphylococcus* bacteriophage' preparation. Antibiotics not reported.	21 days of therapy in one case, further details not reported.	Both infections healed, one in 28 days, the other time to resolution is unclear.	2	0	0	No comment.
Fish *et al.* (2018), [53]<br><br>US<br><br>Case series	2/6<br><br>(4 cases already presented in Fish *et al.*2016).<br><br>All cases were *S. aureus* positive	Chronic non-healing wound	27-year-old female with osteomyelitis on the left great toe refractory to 8 weeks of IV and 2 weeks of oral antibiotics.	No phage sensitivity testing reported.	Monovalent suspension of Staphylococcal phage Sb-1, titre not reported.	Once weekly applications of 0.1-0.5cc. Phage was dripped into the wound cavity, which was packed with phage-soaked gauze, covered with Xeroform® gauze and dry gauze. The phage dressing was left in place for 48h. This patient received 3 applications in 2 weeks before no longer being able to	Reduced inflammation was apparent 14 days after starting phage; the patient did not return to clinic. In June the patient sent a photo showing the wound had healed. Healing was maintained at	1	0	0	'No adverse effects, tissue breakdown or recurrence of infection were seen, and the progression to closure was

	with 'perhaps a second or third organism'.					receive treatment because of caring for a relative.	an unrelated appointment 2.5 years later.				smooth and continuous after initiation of bacteriophage therapy.'
		Diabetic infection	71-year-old female with a toe ulcer. The patient had a *Clostridium difficile* infection secondary to lengthy use of antibiotics and was on vancomycin at the time of presentation for phage therapy. Bone culture revealed *S. epidermidis* and *S. lugdenensis*.			Following debridement, 0.5cc of phage was injected into the distal toe on three occasions: days 1, 7 and 28.	The ulcer resolved in 8 weeks and there was no recurrence at an unrelated appointment approximately 4 months later.	1	0	0	
Fish *et al.* (2018), [13]<br><br>US<br><br>Case report	1/1<br><br>Positive for MSSA.	Diabetic foot infection	63-year-old female with a diabetic foot infection complicated by osteomyelitis.	No phage sensitivity testing reported.	Monovalent suspension of Staphylococcal phage Sb-1, titre not reported.	Injections of 0.7cc of phage around the wound site once weekly for 7 weeks. Levofloxacin was administered after the first 7 days of treatment and was stopped after 7 days because of no notable clinical response. No further antibiotics were given.	Complete cure in 7 weeks. Osteomyelitis remained resolved three years later.	1	0	0	No comment.

Supplementary file four: phage therapy for the treatment of chronic wound or ulcer infections

Gupta *et al.* (2019), [56]<br><br>India<br><br>Case series	20/20<br>Infected with *S. aureus* (n = 5), *E. coli* (n = 6) or *P. aeruginosa* (n = 9).	Chronic non-healing wound	Non-healing wounds of >6 weeks refractory to conventional wound care and systemic antibiotics. Patients were aged 12-60.	Sensitivity confirmed.	A cocktail of three phage isolated from local environmental water sources, each at 10^9 PFU/ml.	The cocktail was applied topically at 0.1ml/cm^2 on alternate days until the wound was bacteriologically sterile.	Bacteriological sterility was achieved after 3 phage applications by day 9 in 9/20 cases and by day 13 in the remaining 11 cases, which received a total of 5 phage applications.<br><br>After three months of therapy 7/20 had complete wound healing. The remaining 13 had significant clinical improvement.	7	13	0	No comment.
Patel *et al.* (2019), [38]<br><br>India<br><br>Case series	46/48*<br><br>*Two patients lost to follow up.<br><br>Polymicrobial infection was present in 13 of 48 cases. Main pathogens were *E. coli* (37.5%), *P. aeruginosa* (31.2%), *S. aureus* (31.2%). Less prevalent pathogens included *Klebsiella*, *Proteus*, *Morganella*, *Citrobacter* and *Acinetobacter*.	Chronic non-healing wound	Non-healing wounds of >6 weeks refractory to conventional wound care and systemic antibiotics. Patient mean age was 47.3. 27/48 were diabetic.	Sensitivity confirmed.	Phage were isolated from local environmental water sources. Customised monovalent or polyvalent preparations were used based on phage sensitivity data. Phages were suspended in 0.9% saline at 10^9 PFU/ml.	Phage preparations were applied using soaked gauze on alternate days at 0.5ml/cm^3, for a total of 5-7 applications, until the wound was bacteriologically sterile.	By day 90 wounds had healed in 90.5% of non-diabetic and 74.1% of diabetic patients.	39	5	2	No comment.
