# Supplementary material for: The Safety and Efficacy of Phage Therapy for Superficial Bacterial Infections: A Systematic Review
Source: Antibiotics (Basel). 2020 Oct 29;9(11):754. doi: 10.3390/antibiotics9110754 (PMC7692203; doi:10.3390/antibiotics9110754)
Supplement: Supplementary file 1 [file antibiotics-09-00754-s001.zip › Supplementary file 5.pdf]

Supplementary file five: phage therapy for the treatment of dermatological infections

Report details		Clinical details						Efficacy				Safety & adverse effects
**Author (year), [citation], location, study type**	**No. of relevant reports and microbiology**	**Condition details**		**Phage sensitivity**	**Phage treatment**	**Treatment schedule and route(s)**	**Outcome**	**Cured**	**Improved**	**No response**		
Larkum (1929), [40]<br>US<br>Case series	231/264<br>All presumed Staphylococcal infection.	Furuncles (n = 208)	Of <1 month [n = 84], 1 month – 1 year [n = 92], >1 year [n = 32]	No phage sensitivity testing reported.	*Staphylococcus* phage lysate.	Subcutaneous (SC) inoculation of 1-2cc once daily. Treatment duration not reported.	77.9% of patients cured without recurrence after >6 months (n = 88), 1.5 to 6 months (n = 61) and <6 weeks (n = 13). Recurrences occurred in 40 patients, and no improvement was reported in 6 patients.	202	0	6		Adverse reactions to SC inoculation of patients infected with *Staphylococcus* with *Staphylococcus* phage lysate were observed in the 'majority of instances' but 'as a rule [were] extremely mild'. Observations were made for 149/208 patients, revealing: no adverse reactions (28.8%); mild local erythema and soreness (34.6%); general, temperature and malaise (7.2%); severe (1%); no report (28.4%).<br>No comment about adverse effects among acne patients.
		Acne (n = 23)	-			Local application. Dose and duration not reported.	Cured, and remained so (8.7%); marked improvement (34.8%); no improvement (56.5%).	2	8	13		
Crutchfield & Stout (1930), [41]<br>US<br>Case series	57/57<br>States a further 62 cases are unreported.	Carbuncles (n = 2)	Of 2-3 days in patients aged 10 and 40	Sensitive, n = 1<br>Not tested, n = 1	*Staphylococcus* phage.	3 and 11 SC injections, supplemented by irrigation.	2/2 recovered.	2	0	0		'Severe local reaction' reported for one case of furunculosis. No other comments on adverse effects.
		Furuncles (n = 13)	Of 2 days to 18 months in patients aged 2 months to 64 years old	Sensitive, n = 3<br>Resistant, n =1<br>Not tested, n = 9		3-21 SC injections, supplemented by topical application (n = 3) or local injection (n = 4).	In 12/13 recovery, discharge or furuncle abortion was reported. In 10/12 cases a recovery	13	0	0		

	All presumed Staphylococcal infection.						time was given, this ranged from 2-14 days, with a mean of 7.3 days. For 1/13 improvement was reported in the first week but recurrence occurred within 9 months.				
		Dermatitis (n = 7)	Of 7 days to 12 months in patients aged 25-45	Sensitive, n = 5 Not tested, n = 2		0-10 SC injections, supplemented by topical application (n = 5) or local injection (n = 1).	Recovery or resolution was reported for 4/7, with improvement seen after 1-2 days of therapy and recovery time of 5-14 days (n = 2). Despite recovery, ‘resistance’ was encountered for 1/4. One patient didn’t improve after 3 injections. For 3/7 improvement was reported on day 3-14, but ‘resistance’ (n = 1) or ‘antiphage’ (n = 1) was encountered.	4	2	1	
		Sycosis vulgaris (n = 8)	Of 1 week to 2 years in patients aged 30-50	Sensitive, n = 3 Not tested, n = 5		0-8 SC injections supplemented by topical application (n = 7).	Recovery, discharge or disappearance of pustules was reported for 8/8, with improvement seen on days 2-4 of treatment and recovery time of 4-14 days (n = 6).	8	0	0	
		Acne (n = 11)	Of 6 months to 8 years in patients aged 15-45	Sensitive, n = 4 Partial, n = 1 Resistant, n = 1 Not tested, n = 5		3-31 SC injections supplemented by local injection (n = 2).	No results were reported for 1/11. Recovery was reported for 4/11, some improvement for 3/11 and no improvement for 2/11. Despite recovery, ‘resistance’ was reported in 2/4.	5	3	2	

		Pyoderma (n = 4)	Of 3 days to 6 weeks in patients aged 4-50	Sensitive, n = 1<br>Partial, n = 1<br>Not tested, n = 2		0-10 SC injections supplemented by topical application (n = 3).	Recovery or discharge was reported for 4/4, with slow improvement. Improvement was from day 2-4 of therapy and recovery time of 3-7 days (n = 2). Despite rapid recovery, ‘resistance’ developed in 1/4.	4	0	0	
		Ecthyma (n = 3)	Of 6 weeks to 2 years in patients aged 25-40	Sensitive, n = 2<br>Not tested, n = 1		0-4 SC injections supplemented by topical application (n = 2).	Recovery was reported in 3/3, with improvement from day 1-2 and recovery time of 7-10 days (n = 2). ‘Resistance’ reported in 2/3.	3	0	0	
		Cellulitis (n = 4)	Of 2 days to 1 year in patients aged 35-50	Sensitive, n = 1<br>Not tested, n = 3		0-10 SC injections supplemented by topical application (n = 3).	Recovery in 4/4, improvement occurred from 0-2 days and recovery time of 3-10 days (n = 2). ‘Resistance’ developed in 1/4.	4	0	0	
		Folliculitis (n = 4)	Of 6 months to 2 years in patients aged 25-50	Sensitive, n = 4		4-10 SC injections supplemented by topical application (n = 1).	Recovery occurred in 2/4, with slight improvement in 1/4 and recurrence in 1/4. Improvements observed from 7-14 days and recovery time of 20 days (n = 1). Despite recovery, ‘resistance’ in 1/4.	3	1	0	

<table>
<tr><td></td><td></td><td>Paronychia
(n = 1)</td><td>Of 1 week in a patient aged 50</td><td>Sensitive, n = 1</td><td></td><td>10 SC injections.</td><td>Slow improvement observed from day 2, discharged after 1 week.</td><td>1</td><td>0</td><td>0</td><td></td></tr>
<tr><td rowspan="8">Beridze et al. (1938), [43], cited in [32]

Georgia

Case series</td><td rowspan="8">143/143

Of 132 cultures isolated 82.5% were S. aureus and 9.8% were mixed Staphylococcal and Streptococcal.</td><td colspan="2">Patients aged 1-60. Infections were acute (1-7 days) or chronic (1-3 years).</td><td rowspan="8">Phage susceptibility screening for all patients implied but outcomes not given. It was noted that negative in vitro susceptibility did not preclude in vivo efficacy likewise susceptibility did not predict efficacy.</td><td rowspan="8">No details reported.</td><td rowspan="8">0.5ml of phage was injected into a cleaned wound and surrounding healthy tissue. The wound was then covered with phage and bandaged. If, on the following day, there was no evidence of irritation, swelling or allergy, phage therapy was continued, with the dose increasing daily to 1ml on day 2, 2ml on day 3. In total 4-5 injections were given. Phage was then stopped and zinc salve or a similar agent was used for 3 days. Phage therapy was restarted if the infection persisted and patients remaining refractory underwent phototherapy.

It is presumed that this or a similar protocol was used for each indication.</td><td>Of 143 patients:
74.8% were successfully treated, improvement was observed in 7.7%, no improvement in 12.6% and the outcome of 4.9% was unknown.</td><td colspan="3">-</td><td rowspan="8">Side effects seen during the first 24-48h after phage administration. The most frequent were ‘fever, malaise, pain at the injection site, sweating, headache, shivering, itching, sleeplessness, bad taste in mouth and weakness.’ These side effects were ‘only temporary and usually followed by a rapid improvement within 24-48h’. The authors consider that phage lysis of bacteria and the consequent release of bacterial proteins may trigger the bodies’ immune system in a manner akin to vaccination.</td></tr>
<tr><td rowspan="3">Furuncles
(n = 73)</td><td rowspan="3">40 acute
13 subacute
20 chronic</td><td>40/40 acute cured.</td><td>40</td><td>0</td><td>0</td></tr>
<tr><td>12/13 subacute cured, 1/13 improved after 20 days.</td><td>12</td><td>1</td><td>0</td></tr>
<tr><td>13/20 chronic cured, 4/20 showed some improvement, 3/20 remained unaffected.</td><td>13</td><td>4</td><td>3</td></tr>
<tr><td colspan="2">Abscesses
(n = 10)</td><td>7/10 cured, 3/10 no improvement.</td><td>7</td><td>0</td><td>3</td></tr>
<tr><td colspan="2">Hidradenitis
(n = 7)</td><td>7/7 cured in 3-5 days.</td><td>7</td><td>0</td><td>0</td></tr>
<tr><td colspan="2">Impetigo contagiosia
(n = 13)</td><td>5/13 cured in 5-6 days.</td><td>5</td><td>?</td><td>?</td></tr>
<tr><td colspan="2">Impetigo vulgaris
(n = 29)</td><td>20/29 cured in an average of 6-10 days, 5/29 no improvement, 4/29 ‘observation ceased and final outcome unknown’.</td><td>20</td><td>?</td><td>5</td></tr>
</table>

		'Various skin diseases' (n = 11)				3/11 cured.	3	?	?	
Bernstein *et al.* (1940), [58], cited in [32]<br>Belarus<br>Case series	141/141<br>No microbiological details.	Furunculosis (n = 119)	No phage sensitivity testing reported.	No details reported.	Phages were administered by SC injection 'no more than 3 times in increasing doses (1ml, 2ml, 3ml) at intervals of 3 days)'. This was supplemented by direct spraying of phages onto the wound at each dressing change.	Outcomes not clearly reported.	Data unclear.			15/141 showed side effects after SC injection. 12/15 were local reactions (redness, swelling) and 3/15 were generalized reactions (fever, rigors).
		Hidradenitis (n = 22)				16/22 cured.	16	?	?	
Izashvili (1957), [57], cited in [32]<br>Georgia<br>Case report	1/1<br>No microbiological details.	Carbunculosis, 75-year old female.	No phage sensitivity testing reported.	No details reported.	0.5ml of phage and penicillin (every 3h) administered subcutaneously on day 1 after presentation. Day 2, the patient was apyrexial. Day 3, a further 1ml of phage was administered subcutaneously. Day 5, patient remained apyrexial. Penicillin was stopped on day 6.	The infection had resolved upon discharge on day 14.	1	0	0	No comment.
Vartepetov (1957), [44], cited in [32]<br>Georgia<br>Case Series	50/50 patients treated in 1937<br>All presumed Staphylococcal infection.	Furunculosis (n = 15); ecthyma vulgaris (n = 2); staphylococcal sycosis (n = 2); folliculitis (n = 6); staphylococcal and streptococcal impetigo (n = 25)	No phage sensitivity testing reported.	Staphylococcal bacteriophages and 'pio-bacteriophages'.	SC injections, 2ml every 24h, supplemented by topical application in some cases.	56% of furunculosis patients were healed.<br>Phage therapy of the other conditions was 'less successful'.	Data unclear.			'Often a local reaction (redness, inflammation) may be observed at the site of phage injection, but this is not an indication that phage treatment should be halted. Sometimes (in about 8% of cases) during phage therapy with Staphylo- or pio-bacteriophages, an

	117/117 patients treated in 1938-41<br><br>All presumed Staphylococcal infection.	Furuncles (n = 47)			25 treated with Staphylococcal phage and 22 treated with 'pio-bacteriophage'.	SC injections at progressively increasing doses (0.5ml, 1ml, 1.5ml and 2ml) every 24h.	60-66% of patients were healed; 13.4-32% of cases showed clinical improvement and 8% of cases showed no effect. Average healing was 4.4 days.	Data unclear.			allergic rash was observed, which rapidly disappeared.'
		Hidradenitis (n = 20); carbunculosis (n = 7); ecthyma (n = 3); sycosis (n = 4); strepto-staphylococcal epidermitis (n = 25); abscesses (n = 4); ulcers & ecthyma (n = 6); folliculitis (n = 1)			Treated with either Staphyloccal phage or 'pio-bacteriophage'.		Data sparse. Average healing for carbunculosis 'about 6 days' and for hidradenitis 7.2 days.	Data unclear.			
Baker (1963), [45]<br><br>US<br><br>Case series	48/56<br><br>All presumed Staphylococcal infection.	Acne vulgaris (n = 34)	Patients aged 11-37	No phage sensitivity testing reported.	*Staphylococcus* phage lysate containing 2 x 10^9 lysed *S. aureus* and $>10^{10}$ 'Gratia' *Staphylococcus* bacteriophage per cc.	0.1cc SC increased by 0.1cc weekly to 0.5-0.6cc. Followed by 0.5-0.6cc every 2-3 weeks for 18-24 months.<br><br>Every SC dose was given with 0.3cc of intranasal aerosol.<br><br>Oral adjunctive ananase (plant protease) given orally.	33 had 'very satisfactory results after 6 months' with 'no new lesions and blanching and thinning of scars.'<br><br>One patient had no relief.	33	0	1	'None of these patients showed any allergic or adverse reaction.'
		Furuncles (n = 14)	Patients aged 3-61		*Staphylococcus* phage lysate (as above).	0.1cc SC and increased by 0.1cc every 3-4 days to 0.3-0.5cc which was then continued once weekly for eight weeks.	'Marked decrease in number, size and tenderness of lesions after the second dose and all patients were symptom free by the third week of treatment and remained so'.	14	0	0	'No systemic reactions' and 'local reactions were mild and did not interfere with treatment'.
Shvelidze (1970), [46], cited in [32]	161/161	Previous antibiotic therapy with penicillin, biomycin and streptomycin was unsuccessful.		No phage sensitivity testing reported.	Anti-Staphylococcal phages.	Anti-Staphylococcal phages were administered using intradermal injections of increasing doses given on	152/161 were successfully treated, significant improvement was recorded	-			No comment.

Georgia<br><br>Case series	139 strains of coagulase positive *Staphylococcus*, 82.7% of which were penicillin resistant.					every second day (0.1ml, 0.2ml, 0.3ml, 0.4ml and 0.5ml) and further continued with a dose of 0.5ml. In total 7-10 injections were given around the infected site.	in 7/161 and no improvement in 2/161. 8.5% relapsed within 3-6 months but completely cured by further phage therapy.				
		Furuncles (n = 62)	Of 3-10 days				97.7% cured<br>3.3% improved<br>0% no effect<br>4.8% re-infected	60	2	0	
		Carbunculosis (n = 54)	Of 5-10 days				66.7% cured<br>20.0% improved<br>13.3% no effect<br>11.1% re-infected	30	17	7	
		Hidradenitis (n = 45)	Of 5-10 days				88.9% cured<br>6.7% improved<br>4.4% no effect<br>6.6% re-infected	37	6	2	
Slopek *et al.* (1987) [33]<br><br>Poland<br><br>Case series	146/550	Furuncles (n = 55)	All antibiotic resistant. 50/55 Staphylococcal infection, 5/55 polymicrobial, all including Staphylococci.	No phage sensitivity testing reported. However, according to (Slopek *et al.* 1983 [60]), sensitivity confirmed, results not shown.	No details. However, according to (Slopek *et al.* 1983 [60]), a library of 259 phages was available for use. Crude phage lysates were used therapeutically.	No details. However, according to (Slopek *et al.* 1983 [60]), suggests that oral and local phage therapy was used.	'Good therapeutic result was obtained in all the cases (100%)'. Two of these cases showed 'marked improvement' and were culture negative.	53	2	0	No comment in this report. However, this report collates previous results which included the comments shown in supplementary file three.
		'Skin inflammations' (n = 91)	83/91 antibiotic resistant. Infections included abscesses, phlegmone, acne. 74 monomicrobial infections: 65/74 *Staphylococcus*; 5/74 *Pseudomonas*; 3/74 *Klebsiella*; 1/74 *Escherichia*.17				The infection was 'eliminated' in 86/91. Four of these cases showed 'marked improvement' and were culture negative. In 5/91 there was 'improvement with tendency towards healing', these were classed as 'transient improvement'.	82	4	5	

			polymicrobial infections.								
Zhvania *et al.* (2017), [54]<br><br>Georgia<br><br>Case report	1/1<br><br>'Affected areas of skin showed strong growth for *S. aureus*'.	Chronic, widespread skin infection.	Netherton Syndrome patient, 16-years old<br><br>Chronic (from 2 months old) antibiotic resistant *S. aureus* infection. Patient allergic to most groups of antibiotics and ointment bases.	Phage used chosen on basis of sensitivity testing.	Three phage products were used:<br>(1) 'Pyo' phage cocktail (vs. *Staphlylococci, Streptococci, E. coli, P. aeruginosa* and *Proteus*)<br>(2) 'Fersis' phage cocktail (vs. *Staphylococci* and *Streptococci*)<br>(3) Sb1 anti-Staphylococcal phage<br><br>All were used at ~10^7 PFU/ml.	20 days of topical application of 'Pyo' phage solution, cream-based application of Sb and once-daily oral (10ml) doses of each of 'Pyo' and Sb1. Oral administration was preceded by 100ml alkaline mineral water. 'Pyo' was also used to treat eye and nose infections.<br><br>Treatment was stopped for 2 weeks (no reason given) and subsequently continued for another 20 days. After 3 months the *S. aureus* had developed resistance to 'Pyo', which was substituted for 'Fersis', which was shown to be active.<br><br>Treatment continued for another three months in the pattern of 20 days therapy, 2 weeks intermission.	Visual improvement on day 7 of therapy.<br><br>Netherton Area Severity Assessment declined from 1.4 to 11.2 (after 3 months) and 7.8 after 6 months.<br><br>Significant regeneration of skin without remission at three-month follow-up.<br><br>Stable at six-month follow-up with continued improvement.	1	0	0	No allergic reactions to phage were observed.<br><br>No clinically significant abnormalities in complete blood cell counts, liver function tests, electrolytes or glucose monitoring.
